# “Interest‐holders”: A new term to replace “stakeholders” in the context of health research and policy

**DOI:** 10.1002/cesm.70007

**Published:** 2024-10-29

**Authors:** Elie A. Akl, Joanne Khabsa, Jennifer Petkovic, Olivia Magwood, Lyubov Lytvyn, Ashley Motilall, Pauline Campbell, Alex Todhunter‐Brown, Holger J. Schünemann, Vivian Welch, Peter Tugwell, Thomas W. Concannon

**Affiliations:** ^1^ Department of Internal Medicine American University of Beirut Beirut Lebanon; ^2^ Department of Health Research Methods, Evidence, and Impact (HEI) McMaster University Hamilton Ontario Canada; ^3^ Clinical Research Institute American University of Beirut Medical Center Beirut Lebanon; ^4^ Bruyere Research Institute University of Ottawa Ottawa Ontario Canada; ^5^ Interdisciplinary School of Health Sciences, Faculty of Health Science University of Ottawa Ottawa Ontario Canada; ^6^ Michael G. DeGroote Cochrane Canada Centre and McMaster GRADE Centre McMaster University Hamilton Ontario Canada; ^7^ WHO Collaborating Center for Infectious Diseases, Research Methods and Recommendations McMaster University Hamilton Ontario Canada; ^8^ Department of Nursing and Community Health Glasgow Caledonian University Glasgow UK; ^9^ Clinical Epidemiology and Research Center (CERC), Humanitas Research Hospital Humanitas University Milano Italy; ^10^ School of Epidemiology and Public Health, Faculty of Medicine University of Ottawa Ottawa Ontario Canada; ^11^ Department of Medicine, Faculty of Medicine University of Ottawa Ottawa Canada; ^12^ Clinical Epidemiology Program Ottawa Hospital Research Institute Ottawa Ontario Canada; ^13^ WHO Collaborating Centre for Knowledge Translation and Health Technology Assessment in Health Equity Bruyère Research Institute Ottawa Ontario Canada; ^14^ The RAND Corporation Santa Monica California USA; ^15^ Tufts Clinical and Translational Science Institute Boston Massachusetts USA

**Keywords:** interest‐holders, patient and public involvement, stakeholders, taxonomy, terminology

## Abstract

**Background:**

Given the colonial connotations of the term “stakeholder”, its continued use may be perceived as disrespectful to Indigenous Peoples. While several groups have introduced alternative terms, each has its own limitations. The objective of this article is to introduce “interest‐holders” as an alternative term to “stakeholders” and describe the discussions underpinning the adoption of the new term by the MuSE Consortium.

**Methods:**

The MuSE Consortium is an international network of over 160 individuals with interest and expertise in different aspects relevant to engagement in research. Members of MuSE explored alternative terms and considered their respective merits and limitations. The deliberations considered the literature on the topic and the results of two consultations with the wider MuSE membership on the alternative terms.

**Results:**

We define “interest‐holders” as groups with legitimate interests in the health issue under consideration. The interests arise and draw their legitimacy from the fact that people from these groups are responsible for or affected by health‐related decisions that can be informed by research evidence.

**Conclusion:**

As groups other than the MuSE Consortium have started to adopt “interest‐holders,” we hope its use will reduce confusion related to the multitude of terms used and convey the intended meaning without any negative connotations.

## 1 INTRODUCTION

In the context of health research, the term “stakeholder” has traditionally been used as an umbrella term to refer to groups who are responsible for or affected by health‐related decisions [1]. Typical stakeholder groups include patients, members of the public, providers, and policymakers [1].

The engagement of stakeholders is crucial in the context of health research, as it increases validity and ensures the relevance of the work to the involved groups [2, 3, 4]. In addition, stakeholder engagement helps with the democratization of the research process by ensuring that public funding for research serves the public interest [5]. From an ethical perspective, engagement acknowledges the rights and interests of stakeholders which aligns with treating people with respect, a principle common to all international research ethics frameworks [6, 7].

However, the term “stakeholder” has colonial connotations. It was historically used to refer to the person who drove a stake into the ground to demarcate the land they were occupying or stealing from Indigenous Peoples [8, 9, 10, 11, 12]. Consequently, there are growing concerns that continued use of the term “stakeholder” may be perceived as disrespectful to Indigenous Peoples.

Several groups have introduced alternative acceptable umbrella terms, including “partner” [8, 11], “knowledge user” [13, 14], “interested parties” [8, 10, 15, 16], and “rights‐holders” [11]. However, none of them applies to all relevant groups and each one has its own limitations (further discussed below).

The MuSE Consortium (formerly the Multi‐Stakeholder Engagement Consortium) is an international network of over 160 individuals with interest and expertise in different aspects relevant to engagement in research [17]. Recognizing these concerns, members of the MuSE Consortium explored alternative terms and considered their respective merits and limitations. The deliberations considered the literature on the topic and the results of two consultations with the wider MuSE membership on the alternative terms. The objective of this article is to introduce the term “interest‐holders” as an alternative to “stakeholders” and describe the discussions underpinning the adoption of the new term by the MuSE Consortium.

## 2 “INTEREST‐HOLDER”

We define “interest‐holders” as groups with legitimate interests in the health issue under consideration. The interests arise and draw their legitimacy from the fact that people from these groups are responsible for or affected by health‐related decisions that can be informed by research evidence [1].

For example, patients' legitimate interests include ensuring a health recommendation accounts for their lived experiences [18]. Another example is to ensure that the outcomes that are prioritized to inform health recommendations are of importance to patients. It is important to note that “interest” does not refer to “curiosity” or “attention” and is not meant to be interpreted in a financial sense. Instead, its intended meaning is closer to the concepts of “stake”, “right,” or “entitlement.” We use the term “interest‐holder representative” to refer to an individual who represents an “interest‐holder group” as part of a specific project. In that context, the use of the term “representative” does not imply the representativeness of the entire group, as we recognize that one individual cannot comprehensively represent an entire group [19, 20].

Considering that “interest‐holders” is defined in relation to “legitimate interest,” one could consider it an attribute that is “inherent” to the group. One implication is that even if others (e.g., researchers) do not acknowledge that interest, or if engagement has not yet occurred, the group still inherently “holds that interest.” Another implication is that as an attribute, the “interest‐holder” status should not be affected by whether a member of the group is interested in being engaged or not (which reflects an attitude as opposed to an attribute). Additionally, “interest‐holder” phonetically resembles the word “stakeholder,” potentially making it easier for people to understand its meaning.

One of the limitations of the term “interest‐holders” is that “interest” may be misinterpreted as a financial term (i.e., return on investment). However, the definition provided above addresses this limitation by directly linking the interest to the concept of health decision‐making. Also, “interest‐holder” may be confused with “conflicts of interest.” While the two terms are distinct, they are related. We previously distinguished between the legitimate (nonconflicting) interests of interest‐holder groups and the conflicts of interests of representatives of interest‐holders [21]. In this context, the “interest” in “interest‐holder” refers to “legitimate interests,” as defined above.

## 3 ALTERNATIVE TERMS CONSIDERED

Members of the MUSE Consortium considered alternative terms, which we list below with the reasons why they were not selected.

*Collaborators or partners* [8, 11]: these terms reflect a relationship (that may or may not be established or may be terminated by the collaborator or partner) as opposed to an attribute. Even if others acknowledge the group as having a legitimate interest, these terms do not apply when engagement is still being considered or is never implemented.

*Decision‐makers*: while this term has an empowering aspect for patients, providers, and policymakers, it does not represent other groups with legitimate interests but who are not decision‐makers per se (e.g., principal investigators). While it aligns with the widely accepted definition of “stakeholders” in terms of “being responsible for health‐related decisions,” it does not cover the concept of “being affected by health‐related decisions.” Furthermore, under the MuSE approach, interest‐holders can be engaged through providing feedback and not necessarily through making decisions [22].

*Knowledge users* [13, 14]: The Canadian Institutes of Health Research (CIHR) defines a knowledge user as “an individual who is likely to be able to use research results to make informed decisions about health policies, programs and/or practices” [14]. This term has a similar limitation as “decision‐makers” as it does not clearly cover the concept of “being affected by health‐related decisions.” Also, it does not represent an inherent attribute where even those who have no access to knowledge or opt not to use it may still hold a legitimate interest in the issue under consideration.

*Interested parties* [8, 10, 15, 16]: this term is related to interest‐holders. One limitation of that term is that it refers to an attitude (of being interested), which an individual might or might not have, and does not portray an attribute that is “inherent” to the group.

*Affected groups* [15, 16]: While the above definition of “interest‐holders” refers to “groups who are affected by health‐related decisions,” the alternative term should ideally help in empowering those groups. The use of “affected” as the alternative does not achieve the goal of empowerment.

*People* [23]: while this term is simple and avoids any negative connotation, it might be too generic and nonspecific to relay the intended concept. The meaning could be easily missed so it would require explanation at every use. In addition, it is not suitable when referring to institutions or organizations (e.g., payers, product‐makers).

*Rights‐holders* [11]: while this term can empower patients, providing “rights” to other categories (e.g., product‐makers) is likely to be considered inappropriate by many. Indeed, right‐holder (e.g., when referring to Indigenous Peoples) has a legal meaning with a stronger connotation than stakeholder.

*No umbrella term*: one suggestion has been to have no umbrella term. We believe that having an umbrella term is important to advance the agenda of engagement of “interest‐holders” in health research and policy. Also, in the absence of better alternatives, people will continue to use suboptimal terms (e.g., stakeholders).

## 4 CONCLUSION

The MuSE Consortium has now adopted the term “interest‐holders” in its publications on engagement in guidelines and in evidence syntheses in the health field. Other groups have also adopted the use of this term [24, 25]. We hope that “interest‐holders” will reduce confusion related to the multitude of terms used and convey the intended meaning without any negative connotations.

## AUTHOR CONTRIBUTIONS

**Elie A. Akl**: Conceptualization; writing—original draft. **Joanne Khabsa**: Conceptualization; writing—original draft. **Jennifer Petkovic**: Conceptualization; writing—review and editing. **Olivia Magwood**: Conceptualization; writing—review and editing. **Lyubov Lytvyn**: Conceptualization; writing—review and editing. **Ashley Motilall**: Conceptualization; writing—review and editing. **Pauline Campbell**: Conceptualization; writing—review and editing. **Alex Todhunter‐Brown**: Conceptualization; writing—review and editing. **Holger J. Schünemann**: Conceptualization; writing—review and editing. **Vivian Welch**: Conceptualization; writing—review and editing. **Peter Tugwell**: Conceptualization; writing—review and editing. **Thomas W. Concannon**: Conceptualization; writing—review and editing.

## CONFLICT OF INTEREST STATEMENT

All authors declare no conflict of interest.

## PEER REVIEW

The peer review history for this article is available at https://www.webofscience.com/api/gateway/wos/peer-review/10.1002/cesm.70007.

## Data Availability

Data sharing not applicable to this article as no datasets were generated or analyzed during the current study.
